# The Epidemiological and Clinical Profiling of Heart Failure—A Retrospective and Comparative Analysis of Cases Before, During, and After the COVID-19 Pandemic in a Romanian Emergency County Clinical Hospital

**DOI:** 10.3390/medicina61112037

**Published:** 2025-11-14

**Authors:** Maria Cristina Tătar, Martin Manole, Iuliu Gabriel Cocuz, Alexandru-Constantin Ioniță

**Affiliations:** 1 Department M3, Clinical Sciences, Faculty of Medicine, “George Emil Palade” University of Medicine, Pharmacy, Science and Technology of Targu Mures, 540142 Targu Mures, Romania; maria.tatar@umfst.ro; 2 Department of Internal Medicine II, Emergency Clinical County Hospital, 540042 Targu Mures, Romania; 3 Faculty of Medicine, “George Emil Palade” University of Medicine, Pharmacy, Science and Technology of Targu Mures, 540142 Targu Mures, Romania; alexionita2@gmail.com; 4 Pathophysiology Department, “George Emil Palade” University of Medicine, Pharmacy, Science and Technology of Targu Mures, 540142 Targu Mures, Romania; iuliu.cocuz@umfst.ro; 5 Clinical Pathology Department, Mures Clinical County Hospital, 540011 Targu Mures, Romania

**Keywords:** heart failure, COVID-19, NT-proBNP, gender specificity

## Abstract

*Background and Objectives*: Heart failure (HF) represents a clinical syndrome characterized by symptoms and signs such as fatigue, dyspnea, edema of the lower limb, or pulmonary rales. It usually occurs in elderly individuals due to decreased cardiac pumping function and/or increased diastolic ventricular filling pressures. The COVID-19 pandemic deeply altered many daily life habits, and one of the most affected groups of people were those with chronic diseases because of their need for regular medical follow-up. Furthermore, SARS-CoV-2 infection itself has been shown to exacerbate cardiovascular diseases (CVDs). *Materials and Methods*: This retrospective, observational, and comparative study aimed to characterize and compare patients with chronic heart failure hospitalized in the Cardiology Department of Medical Clinic II, Mureș County Emergency Clinical Hospital, in Târgu Mureș, Romania, between January and December 2019 (pre-pandemic), January and December 2021 (pandemic), and January and December 2023 (post-pandemic). *Results*: A total of 406 patients were analyzed: 202 patients hospitalized in 2019, 101 patients hospitalized in 2021, and 103 patients hospitalized in 2023. Women with HF were significantly older (median age 72 years) than men (median age 65 years; *p* < 0.001). During the pandemic, the median length of hospitalization increased (8 days vs. 7 days in the other periods). The pandemic period was also associated with a decrease in left ventricular ejection fraction (LVEF), as reflected by a higher incidence of patients with HF with reduced ejection fraction (42% during the pandemic; *p* < 0.01). *Conclusions*: During and after the pandemic, men exhibited significantly higher rates of right and left bundle branch blocks, as well as chronic obliterating artery disease of the lower limb. Left ventricular function declined during the pandemic in both men and women. Throughout the years, we observed distinct patterns between male and female patients regarding associated diseases or behaviours, suggesting lifestyle and psychological changes due to the COVID-19 pandemic.

## 1. Introduction

The COVID-19 pandemic, caused by severe acute respiratory syndrome coronavirus 2 (SARS-CoV-2), was declared on 11 March 2020, by the World Health Organization (WHO) following a rapid global increase in cases [1]. Mortality in COVID-19 often resulted from acute respiratory distress syndrome or from the exacerbation of pre-existing comorbidities, especially cardiovascular diseases (CVDs), which represented one of the most important risk factors for severe or fatal outcomes [2]. Studies conducted in China and Europe showed that up to 40% of hospitalized COVID-19 patients had pre-existing cardiovascular conditions, and many presented elevated cardiac biomarkers, arrhythmias, or heart failure as complications [3].

**Figure 1:**
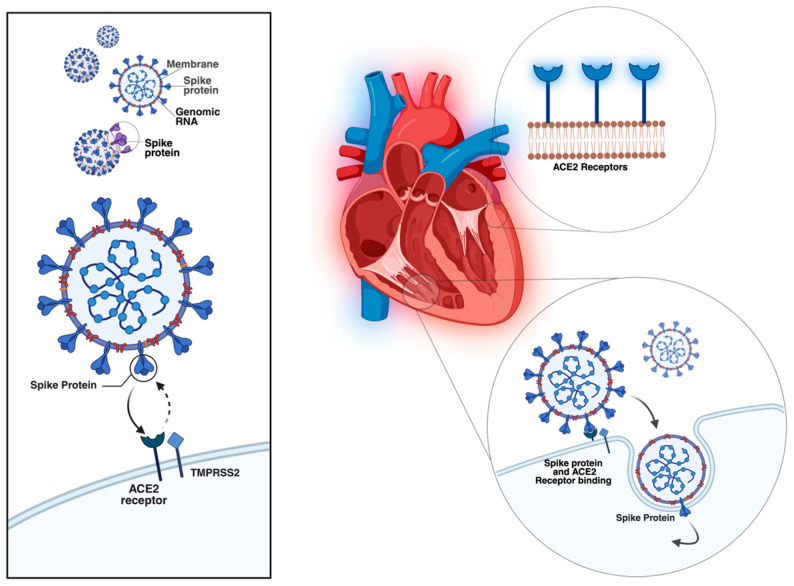
Entry mechanism of SARS-CoV-2 into the cardiac cells (original figure created by Manole Martin with Biorender) [12].

**Figure 2:**
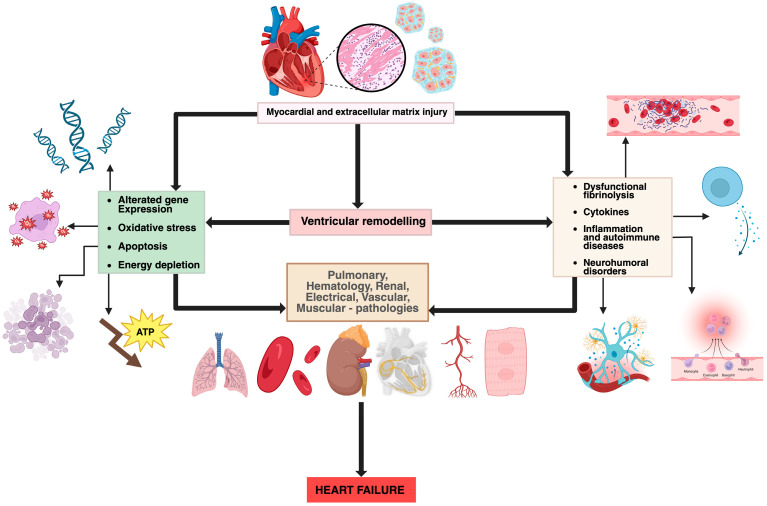
Pathophysiological mechanisms involved in heart failure (original figure created by Manole Martin with BioRender) [13].

Several mechanisms have been proposed to explain the cardiac involvement in COVID-19. Direct myocardial injury occurs through SARS-CoV-2 binding to angiotensin-converting enzyme 2 (ACE2) receptors, highly expressed in myocardial pericytes, which may lead to microcirculatory dysfunction and cardiac remodelling [4]. In addition, indirect injury mechanisms include systemic inflammation, cytokine storm, neutrophil response, hypoxemia, and endothelial dysfunction, all of which can accelerate pre-existing cardiovascular disease and precipitate acute or chronic heart failure [3,5]. The main mechanisms of viral-induced myocardial injury and the pathophysiological processes in heart failure are summarized in Figure 1 and Figure 2, both original illustrations created by the authors using BioRender.

Heart failure (HF) represents a clinical syndrome characterized by typical symptoms such as dyspnea, fatigue, and edema, associated with objective evidence of cardiac dysfunction [6]. It affects approximately 1–3% of the adult population worldwide and more than 10% of individuals aged over 70 years [6,7]. In Europe, prevalence continues to rise due to population ageing and the increasing burden of comorbidities. Romania ranks among the EU countries with the highest rates of heart failure, following Hungary, Italy, and Latvia, as reported by the National Institute of Public Health [7]. The most frequent causes of HF include ischemic heart disease (IHD), hypertension (HTN), valvular heart disease (VHD), cardiomyopathies, and rhythm or conduction disorders [6,8]. Despite therapeutic progress, HF remains associated with high morbidity, frequent hospital readmissions, and an unfavourable long-term prognosis, with five-year mortality rates approaching 50% [9].

The COVID-19 pandemic significantly disrupted healthcare delivery, particularly affecting patients with chronic conditions such as HF. Restrictions, resource reallocation, and fear of infection led to delayed hospital presentations, reduced access to diagnostic procedures, and potential underdiagnosis of decompensated HF. Conversely, the direct cardiovascular effects of SARS-CoV-2, combined with psychosocial stress and limited outpatient monitoring, may have worsened clinical outcomes among these patients [10,11]. While numerous studies have investigated the cardiovascular complications of COVID-19, most have focused on acute infection rather than its indirect and long-term impact on patients with chronic heart failure.

This study provides a longitudinal perspective on the epidemiological and clinical evolution of heart failure patients across the following three distinct timeframes: before, during, and after the COVID-19 pandemic in a Romanian tertiary emergency hospital cardiology clinic. Unlike most existing reports that focus exclusively on the acute impact of COVID-19 on cardiovascular disease, our analysis captures both the immediate and the delayed effects of the pandemic on chronic heart failure management. The inclusion of a post-pandemic cohort (2023) allows for a comparative assessment of recovery patterns and persistent alterations in cardiac function, comorbidities, and hospitalization characteristics. Therefore, the aim of this study was to evaluate changes in the epidemiological and clinical characteristics of patients hospitalized with chronic heart failure before, during, and after the COVID-19 pandemic by comparing data collected from 2019, 2021, and 2023.

## 2. Materials and Methods

This retrospective, observational, and comparative study included patients with chronic heart failure and comorbidities hospitalized in the Cardiology Department of Medical Clinic II, Mureș County Emergency Clinical Hospital, in Târgu Mureș, Mureș County, Romania, during the following three distinct periods: January–December 2019 (before the pandemic), January–December 2021 (pandemic), and January–December 2023 (after the pandemic). This study aimed to characterize temporal changes rather than establish causal relationships regarding the demographic, clinical, and epidemiological characteristics of HF patients across these timeframes. All consecutive adult patients (≥18 years old) with a primary diagnosis of chronic heart failure only from the inpatient clinic were included during each study year, and no convenience sampling was applied. During the pandemic period (2021), fewer patients were hospitalized due to stricter admission criteria: only those presenting with a negative SARS-CoV-2 test were admitted for non-emergency hospitalization. Data were collected from patient observation sheets, discharge letters, test reports, and investigations performed during hospitalization.

HF severity was assessed using both the New York Heart Association (NYHA) functional classification (I-IV) and left ventricular ejection fraction (LVEF): heart failure with preserved ejection fraction (HFpEF), heart failure with mildly reduced ejection fraction (HFmrEF), and heart failure with reduced ejection fraction (HFrEF). LVEF was measured by transthoracic echocardiography, calculated in percentage (%), and determined using the following techniques: modified Simpson’s biplane method, visual estimation method and Teichholz method. Measurements were performed by different physicians; Simpons’s biplane method was primarily used, while the visual estimation or Teichholz method were performed only when the image quality was suboptimal. However, we could not state which method was used for every patient.

Nutritional status was assessed using only body mass index (BMI) WHO classification.

The NT-proBNP value was measured and considered in this study because it correlates with the prognosis of patients with cardiovascular diseases such as heart failure, VHD, and coronary artery disease (CAD). Normal values were considered according to age as follows:

- <125 pg/mL for patients under 75 years of age
- <450 pg/mL for those over 75 years of age.

Comorbidities refer to associated diseases or risk factors such as smoker status.

The category “Valvular heart disease” included the following:

- At least one valvular heart disease of at least second degree (mild or higher), demonstrated by transthoracic or transoesophageal echocardiography.
- Prosthetic valve of any type.
- Physiological traces were not considered for this category.

The patients were not stratified in accordance with the severity of their condition.

Individual COVID-19 infection data were not available; therefore, pandemic-related changes were interpreted as temporal associations rather than direct viral effects. Moreover, many patients likely had asymptomatic forms of COVID-19, which made the causal analysis less relevant, since we cannot know precisely which patient had or did not have the disease.

Sex-stratified and mortality analyses were performed as secondary exploratory analyses and as a part of the epidemiological characteristics.

The following exclusion criteria were applied to ensure cohort homogeneity and minimize confounding: genetic syndromes with cardiovascular involvement, as these represent distinct pathophysiological entities, and history or current malignancy (except basal cell carcinoma), given the potential effects on inflammatory markers and cardiac function and autoimmune disorders due to their association with chronic inflammation (Table 1).

### Statistical Analysis and Figure Design

A database was created using Microsoft Excel version 16.62 that included anthropometric and demographic variables, clinical and paraclinical parameters, comorbidities, length of hospital stay, and in-hospital mortality. Statistical analysis, tests, and graphs were performed using GraphPad Prism 10 (v10.4.2).

All statistical tests used were two-tailed, and significance was set at *p* < 0.05, with a 95% confidence interval (CI). For continuous variables, the distribution type was assessed using the Shapiro–Wilk test. Parametric variables are expressed as mean ± standard deviation (SD), while non-parametric variables are expressed as median and interquartile range [IQR]. The following tests were used as appropriate: ANOVA, Kruskal–Wallis, Mann–Whitney, and Chi-square. Figures from the Introduction section were generated with BioRender and are original. Multiple *p*-values were adjusted with Bonferroni correction when appropriate. All analyses were exploratory and unadjusted for confounders, given the descriptive nature of the study.

## 3. Results

### 3.1. Cohort General Characteristics

Within this study, data were collected from 406 patients aged between 36 and 96 years old. The study seeks to characterize outcomes in individuals with chronic heart failure. A total of 202 patients from 2019 (pre-pandemic), 101 patients from 2021 (pandemic), and 103 patients from 2023 (post-pandemic) were included in this analysis. A secondary gender-stratification analysis is added in every subsection and is not treated individually, as it represents an important epidemiological parameter.

Table 2 shows data and statistical analysis regarding the age and nutritional status of the patients from the entire cohort. The gender distribution revealed a slight male predominance across the entire cohort, with 241 men (59.4%) and 165 women (40.6%). The median age of the entire cohort was 69, with male patients having a median (66 years) lower than female patients (median = 72 years) suggesting that women develop heart failure later in life than men. Throughout each study period, the mean age remained relatively stable without significant differences (*p* > 0.05). In the entire group of patients, a small but insignificant difference between the genders was identified (*p* > 0.05), with both being at the limit for the first grade of obesity (Table 2).

**Figure 3:**
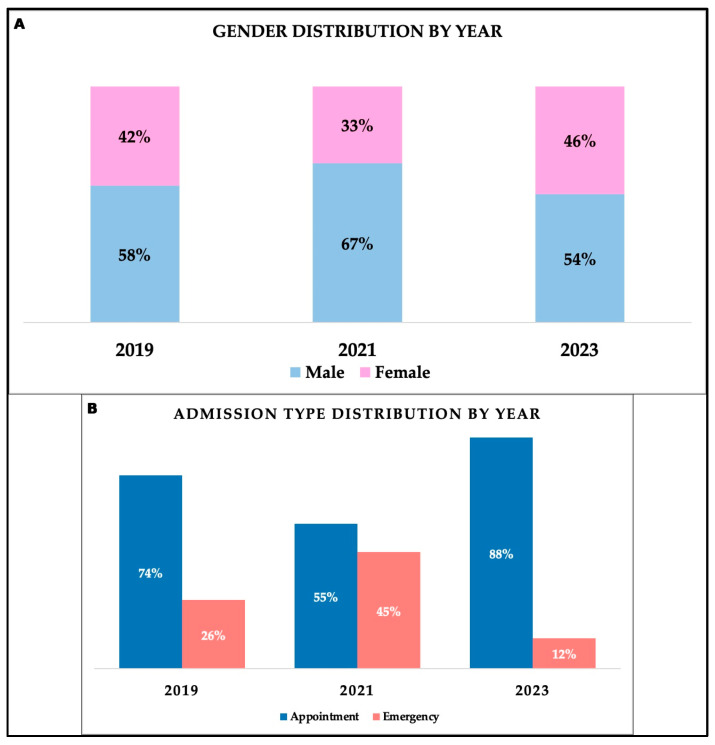
Comparative analysis by year of patients’ characteristics. (**A**). Gender distribution by year. (**B**). Admission type distribution by year.

Table 3 and Figure 3 show the comparative analysis of different variables and general characteristics, highlighting the differences throughout periods. Overall, gender distribution remained relatively constant, with a slight male predominance. However, a decrease in the proportion of women by 32.67% was observed in 2021. Also, a rise in emergency admissions during the pandemic period was observed, and in 2023, the trend was descending, as shown in the Figure 3. From one year to another, there were small insignificant differences regarding the age of men and women in different periods (men *p* = 0.923; women *p* = 0.52), with most patients being between 60 and 79 years old, a trend maintained throughout the years. The BMI values showed constant median values and were not affected significantly by different periods.

**Figure 4:**
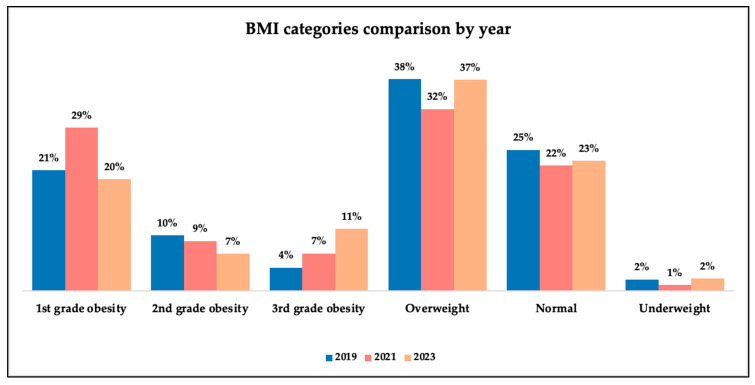
BMI category comparison graphical analysis.

Figure 4 illustrates the distribution of BMI categories across the study years. Most patients were classified as overweight. We noted a decrease regarding the incidence of overweight patients in the pandemic period, at the same time as a rise in patients with the first grade of obesity in the same year. Proportions remained relatively stable for the other categories throughout the different periods.

### 3.2. Functional Status (NYHA Class) and Left Ventricular Function (LVEF)

Table 4 summarizes the NYHA functional class and LVEF categories across the entire cohort. A predominance of NYHA class III patients was observed, while NYHA class I cases were rare. Regarding ejection fraction, HFpEF was the most frequent phenotype (54%), followed by patients with HFrEF (33%) and HFmrEF (13%).

Table 5 summarizes an extensive comparative analysis of the NYHA classes’ distribution in relation to different variables such as gender, environment of origin, and admission type. Significant differences were observed constantly throughout the different periods regarding the admission type, as follows: less symptomatic patients (NYHA class II) were admitted mostly through appointment admissions (2019, n = 65; 2021, n = 15; 2023, n = 32) and those from severe classes (NYHA class IV) were admitted mostly through the emergency department (2019, n = 9; 2021, n = 21; 2023, n = 3), usually as a decompensation episode. The environment of origin and gender did not suggest significant differences regarding symptoms and indicate that these parameters do not alter symptoms that are subjective clinical indicators of HF severity.

**Figure 5:**
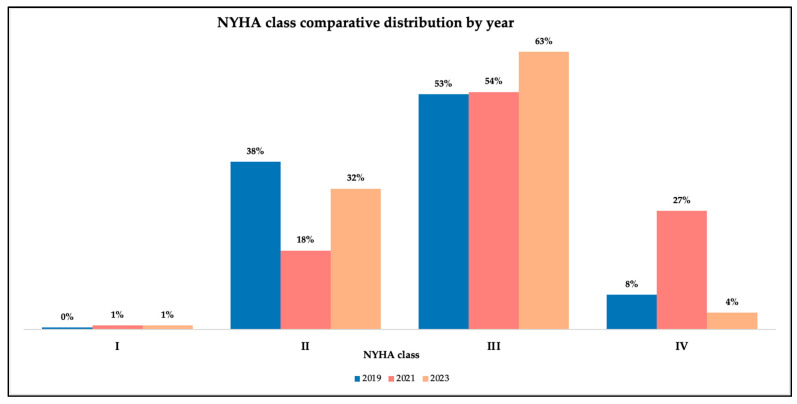
NYHA class comparative distribution through the studied periods.

Figure 5 displays a comprehensive comparison analysis through the years regarding NYHA classes’ distribution. We observed that most of the patients were classified as class III in every period (2019, n = 108; 2021, n = 54; 2023, n = 65). Also, we noticed a rise in NYHA class IV during the pandemic (n = 27), with a decline after the pandemic period (n = 4).

**Figure 6:**
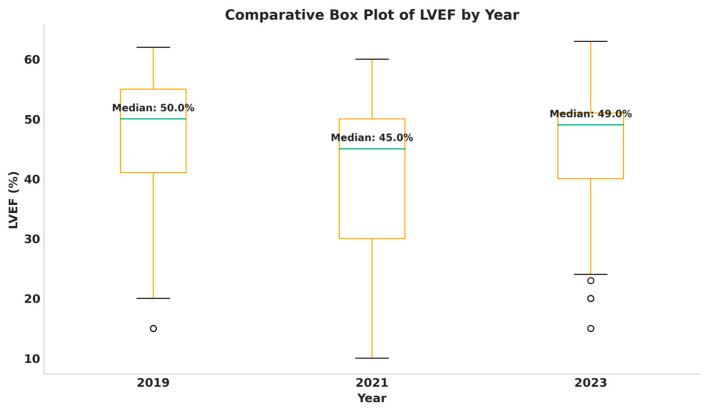
Graphical comparative analysis of LVEF values by year.

Figure 6 shows a comparative analysis of LVEF values by year. We found an important decrease in LVEF in patients from the pandemic period, from a median of 50% in 2019 to a median of 45% in 2021, and a rise in these values after the pandemic, with values of 49%, suggesting more compensated patients.

Table 6 shows that LVEF presented gender-specific differences. Across the cohort, women presented higher LVEF values (median = 50%, mean = 47.02 ± 10.31) compared to men, who had a 3% lower median and a considerably lower mean (43.31 ± 12.24). Significant differences were also noted by the type of hospital admission, with emergency admissions showing median LVEF values as much as 10% lower than scheduled admissions. Temporal analysis revealed a decline in LVEF during the pandemic, with a return of median values to the pre-pandemic level in 2023. At the same time, in 2021, patients presented a greater variability of LVEF, and in 2023, the values were in a much narrower range. Gender differences were significant only in 2023 (*p* = 0.0116), with men having an average of 41% ejection fraction (median = 45%), and women fared better from this point of view, with an average LVEF of 47.5% (median = 50%). The distribution of LVEF values according to the type of hospitalization showed that, prior to the COVID-19 pandemic, the highest ejection fraction values were found, in contrast to the pandemic period when the lowest values were recorded. Specifically, the median LVEF among patients admitted through the emergency department was 40%, compared to 50% among those admitted on scheduled basis. In 2023, both types of patients (appointment and emergency admissions) returned to baseline levels of left ventricular contractile function, accompanied by a lower variability compared with previous years.

**Figure 7:**
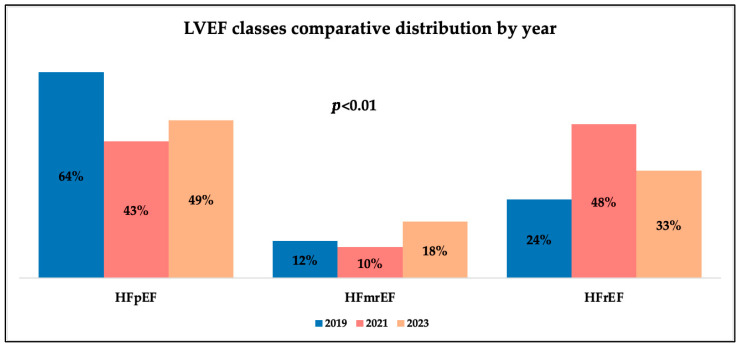
Graphical comparative analysis of LVEF classes by year.

Figure 7 shows the analysis and distribution of HF types regarding LVEF categories and suggests important differences regarding time trends. We found significant fluctuations (*p* = 0.0014) in the proportions regarding LVEF classes, as follows: the incidence of patients with HFrEF increased during the pandemic (42%), only to slightly decrease after the pandemic (27%). In contrast, HFpEF showed a decrease in the percentage of patients in 2021 (47.6%) compared to 2019 (68.3%), with a recovery in 2023 (54%). As for the HFmrEF class, there were relatively stable proportions in 2019 (12%) and 2021 (11%) and a slightly higher rate of these patients in the post-pandemic period (19%).

### 3.3. Length of Hospital Stay and Its Association with NYHA Class

Table 7 offers a comparative analysis of length of stay in the hospital by gender each year. We observed that the average length of hospitalization, reported for the entire group, reached approximately 9 days, with a negligible difference between women and men. However, temporal variations were observed: the median length of stay increased in 2021 (8.0 [6.0–12.0] days) compared to 2019 (7.0 [5.0–9.25] days), which subsequently decreased post-pandemic (7.0 [5.0–10.0] days). The significant differences were between 2019 and 2021 (*p* = 0.0014) and between 2021 and 2023 (*p* = 0.0058). We did not find significant differences regarding length of stay between genders.

Table 8 presents a statistical analysis of NYHA classes in relation to length of stay each year through associations, correlations, and regression tests. We explored the relation between NYHA class and the length of hospitalization, demonstrating a weak correlation between the variables in 2019 (*r* = 0.17, *p* = 0.014), which strengthened to moderate in 2021 (*r* = 0.36, *p* < 0.001) and moderate–strong in 2023 (*r* = 0.42, *p* < 0.001). These results suggest a consistent link between NYHA classes and prolonged hospitalization, with patients of a higher NYHA functional class requiring medical attention for a longer period. At the same time, the correlation coefficient increased from one year to the next, which supports the better predictability, given by the NYHA class, for the increase in hospitalizations depending on functional status. Considering the type of NYHA variables and the duration of hospitalization in Table 8, a quantile regression model was calculated, and the results were significant for 2021 (*β*_1_ = 2.00, *p* = 0.010) and 2023 (*β*_1_ = 3.00, *p* < 0.001), corresponding to an additional 2 days of hospitalization per NYHA class in 2021 and 3 days in 2023, respectively, with the strongest association observed after the pandemic (*p* < 0.001).

### 3.4. The Analysis of Associated Diseases

Table 9 shows the statistical association between associated diseases and in-hospital death regarding the entire cohort. Among all associated conditions, VHD was the only comorbidity significantly associated with lower in-hospital mortality, suggesting a potential protective factor. However, since we did not stratify the patients according to the severity status of their VHD, therefore, we cannot conclude that it represents a protective factor or an indicator of mildly affected forms of HF.

Comparative analysis from Table 10 across the years reveals notable trends regarding the associated diseases. Angina pectoris was more frequent pre-pandemic and declined thereafter (*p* = 0.0347). Atherosclerosis decreased in incidence during the pandemic but increased again in 2023 (*p* = 0.0177). RBBB and LBBB incidence peaked during the pandemic and then experienced a decreasing trend, but still with higher rates than in 2019 (*p* < 0.001 in both cases). PH incidence declined significantly after the pandemic (*p* = 0.0176). In the case of angina pectoris, it has been shown in previous studies that patients with such conditions avoided presenting to the hospital due to fears of infection with SARS-CoV-2 [14]. Atherosclerosis’ lower rate in 2021 may be due either to the limitation of investigations during the pandemic, causing a false decrease due to underdiagnosis, or by a decrease in patients’ presentation even though differences were significant (*p* = 0.0034). Intraventricular conduction disorders (LBBB and RBBB) were present in half of the patients during the pandemic.

Gender-stratified analysis of comorbidities from Table 11 revealed that men were significantly more likely to present with COALL (*p* = 0.0205), RBBB (*p* < 0.001), and/or LBBB (*p* < 0.001). In men, HTN also showed a decreasing trend (*p* < 0.001). By contrast, women exhibited more dynamic changes in cardiovascular comorbidities across the study period. Specifically, the incidence of angina pectoris significantly decreased during and after the pandemic (*p* = 0.001) while ventricular and supraventricular arrythmias showed a non-significant increase during the pandemic but became significantly more prevalent in 2023 (*p* = 0.0221). COALL decreased by 58% in 2021 (*p* = 0.0116), and in 2023, it had an increased rate that exceeded the 2019 level. Chronic venous disease declined in women during the pandemic (*p* = 0.0013), but later it returned to 2019 levels. VHD had a downward trend in female patients in 2021 (*p* < 0.001), followed by a slight increase beyond the 2019 rate in 2023. The IHD rates decreased in 2023 with almost 10% (*p* < 0.001, Table 10). PH decreased constantly in 2021 and 2023 compared to 2019 (*p* < 0.001). Similarly, AF/atrial flutter showed a slight decrease during the pandemic but reached higher post-pandemic rates among women (*p* < 0.001). HTN and hypertensive heart disease were more frequent in women during the pandemic but subsequently decreased (*p* < 0.001). We observed higher rates among men regarding intraventricular conduction disorders: RBBB (21%) and LBBB (27%).

From a gender perspective, Table 12 shows significant differences in women with CKD, who were strongly underrepresented during the pandemic but returned to 2019 levels post-pandemic (*p* = 0.0087). The percentage of female smokers declined progressively over the study period (*p* = 0.0224). The global analysis of risk factors and comorbidities revealed further temporal associations. In 2019, VHD occurred together with atherosclerosis, COALL, and angina pectoris, while anemia was associated with PH, AF/atrial flutter, and atherosclerosis, which highlights the contribution of hematologic abnormalities to the progression of cardiovascular diseases. In 2021, myocardial infarction (MI) was linked to CKD, emphasizing the interplay between renal and cardiovascular injury in the context of COVID-19, a condition with a known inflammatory and thrombotic profile. In 2023, the association of CKD and anemia was prominent, suggesting a subset of patients with advanced HF and renal disease. Throughout all the study years, the classical association between chronic obstructive pulmonary disease (COPD) and smoking persisted.

We discovered an association between HFrEF patients and their main comorbidities. The analysis suggested the following key findings: in 2019, HFrEF demonstrated a significant association with MI (*p* = 0.001) and with ventricular or/and supraventricular arrhythmias (*p* = 0.022). In 2021, LBBB was significantly associated with HFrEF (*p* = 0.0102), and this association persisted post-pandemic (*p* < 0.001). Furthermore, the relationship between HFrEF and MI was reaffirmed in 2021 (*p* = 0.0068), and in 2023, a significant association with ventricular and/or supraventricular arrhythmias was again observed (*p* = 0.0049).

### 3.5. NT-proBNP Levels and the Relation to LVEF and Demographics

Table 13 shows the analysis of NT-proBNP values by year in relation to gender, admission type, place of origin, and age, and it suggests important differences between male and female patients before the pandemic, between rural and urban residents, where there were also differences after the pandemic, and admission type, the analysis of which showed constant differences, as did the age analysis. Natriuretic peptide values were significantly higher (*p*(2019) < 0.001, *p*(2023) = 0.015) in the group of patients over 75 years of age (including 75 years) in 2019 (median = 6653.5 pg/mL) and in 2023 (median = 1955 pg/mL). These findings are consistent with a large cohort study demonstrating significantly higher NT-proBNP levels in patients aged over 77 years old compared to younger individuals [15]. Notably, in 2021, patients admitted electively exhibited significantly higher median (1126.5 pg/mL, Table 13) NT-proBNP values compared to the median in 2019 (407 pg/mL, Table 13). A downward trend in of NT-proBNP values was observed across the study years, culminating in convergence between male and female levels in 2023. The patients’ place of origin also influenced NT-proBNP values in 2023, with rural residents exhibiting higher median values (1846 pg/mL) compared to urban residents (533 pg/mL).

Table 14 shows the results of linear regression analysis between NT-proBNP values and LVEF values by year. The relationship between LVEF and (log) NT-proBNP was examined using linear regression. In 2019, a one logarithmic unit increase in NT-proBNP (approximately a 2.7-fold absolute increase) was associated with a reduction in LVEF by 2.15%. In 2021, the corresponding decrease was 3.39%, and in 2023, it was 1.69%.

### 3.6. In-Hospital Mortality

Table 15 shows the comparative in-hospital mortality rates. We observed a general in-hospital mortality rate of 4% before and after the pandemic, at the same time as a peak mortality rate during the pandemic (14%). Females exhibited a tendency to have higher mortality rates compared to men. We did not dig further into mortality analysis, as it is not one of the main study aims.

## 4. Discussion

Demographic analysis demonstrated male predominance, and regarding age distribution, significant differences between genders were observed within the entire cohort, with men exhibiting a lower median age (*p* < 0.001). This difference was also demonstrated in the Hillingdon study and in the Framingham study, which reported a lower incidence in women aged 50–59 years [16,17]. Similarly, a large South Korean study including approximately 8800 patients with HF found that women had a mean age of 71.1 years and men had a mean age of approximately 5 years younger [18]. As a comparison, females in our study had a median age of 72 years. Patients’ distribution by environment of origin was relatively balanced, with 205 patients originating from rural areas and 201 from urban areas, a pattern consistent across study years. Admission was either by appointment (72.91%) or through the emergency department (27.09%). During the pandemic period, significant differences regarding admission type were observed (*p* < 0.001), as suggested by an important rise in emergency admissions in patients during the pandemic (44.6% admitted through emergency department) compared to 26.2% admitted through emergency services in 2019. Following the pandemic, emergency admissions declined substantially to only 11.7%. A study from Romania that assessed the impact of the pandemic on the management of mental health services supports a general decrease in the number of patients who presented to the hospital during the pandemic [10]. Thus, the fear of being infected with the SARS-CoV-2 virus also represented a negative psychosocial factor, independent of the healthcare system and medical staff, on the type of hospitalizations [10].

Our analysis showed a rise in hospitalization stay during the pandemic period, likely reflecting the reorganization of the public health system during the pandemic and the prioritization of emergency cases. An epidemiological study published in a Romanian scientific magazine examining heart failure hospitalization between 2013 and 2023 reported a national average of hospital stay of 6.5 days [11]. In contrast, we observed longer hospitalizations, and this can be explained by the sample size (smaller number of patients in the present study), but also by a different regional distribution, with Mureș County having more than double admissions for HF patients compared to other regions of the country [11]. These differences may also reflect the role of Târgu Mureș as a referral centre with advanced university-based management of patients for advanced or terminal stages of HF; nevertheless, data may reflect regional biases, as our data derive from a single tertiary unit. In 2022, another study that analyzed the impact of heart failure on the European continent was published by the European Society of Cardiology, demonstrating an average duration of hospitalization of the included countries of 8.5 days [19]. The same article mentions that there is a wide variation in the length of hospitalization from country to country, with Romania reporting an average length of hospitalization around 7 days, most likely due to different diagnostic and management resources and protocols [19].

We found a strong, progressively increasing association between symptom severity and length of stay in the hospital, particularly during and after the pandemic. A study during the pandemic on patients with HF and COVID-19 supported longer hospital stays for patients with more severe symptoms, with a median of 20 days for those in NYHA class IV and 10 days for patients in NYHA I, supporting an association between higher NYHA classes and prolonged hospital stay [20]. Another study that retrospectively observed mortality and hospital stay in patients with HF and LVEF higher than 45% demonstrated increased rates of these indicators for patients who were in NYHA classes III and IV [21]. In our study, ordinal logistic regression confirmed these findings, with OR indicating a 7.7% increase in the probability of a higher NYHA class per additional hospitalization day in 2019 (OR = 1.077, *p* = 0.0136), rising to 11.6% during the pandemic (OR = 1.116, *p* = 0.0032) and returning to 7.8% post-pandemic (OR = 1.078, *p* = 0.0018). These trends likely reflect both the higher clinical severity of patients from the pandemic period and the subjectivity inherent in NYHA classification.

Analyzing patients’ LVEF values, we observed different distributions of LVEF classes by year and gender. We observed that men had significantly lower LVEF values and emergency-admitted patients also exhibited lower values, with a difference of 10% compared to appointment admissions. As has been shown, emergency admissions saw a rise during the pandemic, and alongside this, we observed a decrease in LVEF values throughout the same timeframe. These trends mirror results from recent medical studies, which support an increase in the proportion of patients with lower systolic function during the pandemic, associated with a decrease in those with preserved systolic function and stability in the percentage of patients in the intermediate class over time [22,23,24]. A study focusing on the influence of gender on cardiac function in patients with severe forms of COVID-19 concluded that there were no notable differences, although the majority were male [25]. The explanation for the fact that LVEF is improved in female patients may suggest a better recovery in the female population after the pandemic or more severe, potentially irreversible, damage to men due to the pandemic. It may also imply the presence of other risk factors that have an increased prevalence among males. Associations between HF phenotypes and demographic variables also revealed interesting patterns. HFrEF was significantly associated with male gender in 2019 (*p* = 0.0097), but subsequently it was evenly distributed between men and women post-pandemic. The results are consistent with other studies demonstrating this connection, which mainly support that women with HFrEF have a better survival rate compared to men in the same category and show that premenopausal women most frequently present HFpEF [26,27,28]. Literature further suggests that historical male predominance has diminished over time [27]. When interpreting these findings, it is important to consider that during the pandemic, we had many epidemiological restrictions that could alter the type of patients. In Romania, only patients who presented a negative status of SARS-CoV-2 infection were admitted for elective investigations or checkups, while emergency admissions involved patients with severe conditions, as quarantine or isolation protocols had to be followed. A study focusing on the influence of gender on cardiac function in patients with severe forms of COVID-19 concluded that there were no notable differences, although the majority were male [25].

Regarding patients’ comorbidities, we observed a few classic patterns in accordance with HF physiopathology. These findings mirror the literature, which identifies HTN and CAD/IHD as the primary comorbidities of HF [29]. PH has also been associated with this, along with diabetes mellitus and atrial fibrillation, especially in patients with HFpEF [30]. Conduction disorders observed during the pandemic may reflect ventricular remodelling in advanced HF, exacerbated by systemic inflammatory response and incomplete cardiac recovery in previously affected individuals. VHD was the only comorbidity significantly negatively associated with mortality, in fact being a protective factor. Even though this condition is a known predictor of one-year mortality and rehospitalization, particularly when combined with PH [31], we could not interpret a strong causality effect, as we did not stratify the patients in accordance with VHD severity. Also, these results may be altered by the small groups implied in the statistical calculation, as well as reflecting a regional bias. Studies suggests that patients with a lower severity of VHD may have a better survival rate, and we may have had to deal with a high prevalence of less severe or surgically treated cases of VHD, altering the results [32,33].

Atherosclerosis and LBBB showed increased incidence during the pandemic, aligning with other studies where LBBB was associated with mortality rates [34]. Side effects of certain treatment for COVID-19 have also been described as being responsible for increased rates of intraventricular conduction disorders [35]. Referrals for the diagnosis of PH and lower incidence rates during and post-pandemic have been reported internationally [36,37]. These findings are consistent with the existing literature, identifying HTN, IHD, AF, arrythmias, and MI as major comorbidities and aggravating factors among patients with HFrEF [38,39]. We additionally observed significant increases in RBBB and LBBB incidence, especially in men (*p* < 0.001 for both disorders). Such patterns reported in other studies may be linked to the systematic inflammatory and cardiac damage induced by COVID-19, which could have promoted ventricular remodelling and delayed post-pandemic cardiac recovery in HF patients [34,35].

Among non-cardiovascular comorbidities and risk factors, type 2 diabetes mellitus, chronic kidney disease (CKD), and smoker status were most prevalent. These comorbidities are recognized as major contributors to HF progression, especially in patients with HFrEF, being correlated with longer hospitalizations and higher mortality rates [40,41]. Anemia, frequently observed in CKD, is primarily attributable to erythropoietin deficiency and disordered iron metabolism [42]. COPD and smoking are well established to be causally linked, with smoking accounting for approximately 70% of COPD cases [43].

The analysis of NT-proBNP values revealed significantly higher values among women in 2019, alongside inter-annual differences with each gender. Among men, significant differences were noted between 2019 and 2023 (*p* = 0.041), with higher median NT-proBNP levels in 2019 (2132 pg/mL) and between 2021 and 2023 (*p* = 0.003), and lower values in 2023 (665 pg/mL). For women, significant differences were identified between 2019 and 2023 (*p* = 0.003), with higher median values in 2019 (3983 pg/mL) and between 2021 and 2023 (*p* = 0.037), with elevated median values during the pandemic (2747 pg/mL). Prior studies similarly reported higher NT-proBNP concentrations in women, which may indicate gender-specific baselines or support the rationale for gender-tailored therapeutic approaches in the future [44,45]. Admission type also influenced NT-proBNP levels each year, with emergency patients presenting significantly higher median levels (5300–5600 pg/mL) each year compared to those from scheduled admissions (400–1200 pg/mL). This pattern may reflect the heightened frequency and severity of cardiac decompensations during the pandemic due to impaired oxygenation and a pro-inflammatory state, both of which increase thrombotic risk with supplementary heart damage [46], as well as psychosocial factors.

Finally, in-hospital mortality peaked during the pandemic, consistent with other studies reporting an excess mortality rate of approximately +4.5% during the pandemic compared to the expected levels [47,48], while other researchers support higher mortality rates exceeding 10%, but with smaller samples [48]. A French study encompassing 2.7 million acute hospitalizations reported a peak in-hospital mortality of 13% in 2022, with a subsequent 50% decrease in 2023 [11]. As for Romania, the national in-hospital mortality rate for heart failure patients is around 5% and does not exceed 10%, which is consistent with the findings of the present study [11]. These findings cannot imply causality by the nature of the study design and data collection.

### Study Limitations

This was a retrospective study, and, therefore, variables of interest and potential confounding factors could not be entirely controlled. Moreover, all data were collected from a single Romanian tertiary cardiovascular centre (the Mureș County Emergency Clinical Hospital), which may introduce geographical or cluster bias. Body mass index (BMI) was calculated only for those patients whose records contained the necessary variables, potentially limiting representativeness. Similarly, NT-proBNP values were not available for all patients, which may affect comparability. LVEF measurements were not standardized and were operator-dependent, potentially introducing bias or measurement error, as they were obtained using different methods, by different operators, and on different devices.

Additionally, for certain cardiac conditions, standardized quantitative data on relevant risk exposures (e.g., dietary patterns, smoking habits, alcohol consumption, and treatment adherence) were not systematically recorded or quantified. Collectively, these limitations may reduce the statistical power of the analysis; nevertheless, they underscore the need for further research into how heart failure is diagnosed and treated in the post-pandemic period and how strategies should be tailored according to gender differences.

Consistent information regarding COVID-19 infection or vaccination status was not available for most patients. Therefore, the findings could not be interpreted as evidence of causal relationship with COVID-19, but rather as an observation of temporal changes across the three studied periods.

## 5. Conclusions

This retrospective and comparative analysis provides an integrated overview of the clinical and epidemiological evolution of chronic heart failure across pre-pandemic, pandemic, and post-pandemic periods in a Romanian tertiary care hospital clinic.

Before the COVID-19 pandemic, distinct epidemiological, presentation, and management characteristics of heart failure were observed. These patterns were profoundly altered throughout the pandemic. This milestone was a stress factor for patients and healthcare systems around the world, but especially in Romania, disrupting access to care, producing surges in emergency admissions, leading to longer and more heterogenous hospitalization, and causing higher in-hospital mortality. There were changes in heart failure presentation—particularly a rise in the incidence of HFrEF and a decline in HFpEF—along with a reduction in LVEF values, highlighting the blended impact of delayed presentations and more severe decompensation of these chronic patients. Although data from after the pandemic suggested a partial recovery in scheduled care, diagnostic rates, and functional status, persistent arrhythmias, conduction disorders, and comorbidity rebounds emphasize the long-term consequences of the pandemic and epidemiological restrictions, not only as a medical condition, but as a psychosocial disruptor

Differences related to gender were clear through the following results: women presented at older ages, maintained higher LVEF values, and demonstrated specific comorbidity dynamics, whereas men showed higher burdens of conduction disorders. Biomarker analyses confirmed the prognostic value of NT-proBNP and reinforce the differences regarding gender, advanced age (patients over 75 years old), and admission type. These results reiterate the relevance of integrating this investigation as an ordinary test for heart failure patients’ status assessment and the fundamental importance of developing gender and heart failure stage stratification values for these markers. Also, pandemics or other exacerbating factors for heart failure patients should be taken into consideration for refining and developing more accurate biomarkers in patients’ assessment and management.

## Figures and Tables

**Table 1:** Inclusion and exclusion criteria.

Inclusion Criteria	Exclusion Criteria
Adults (≥18 years) diagnosed with chronic heart failure	Genetic syndromes with cardiovascular involvement
Hospitalized in the inpatient Cardiology Department of Medical Clinic II of the Mureș County Emergency Clinical Hospital, Târgu Mureș, Romania	History or current diagnosis of malignancy, with or without treatment (except basal cell carcinoma)
Admitted during the following periods: January 2019–December 2019, January 2021–December 2021, and January 2023–December 2023	Autoimmune disorders with or without treatment

**Table 2:** Statistical analysis of the entire cohort anthropometric characteristics.

Variables	Gender	n	Median [IQR]	*p*
Age (years)	Male	241	66.0 [60.0–74.0]	<0.001
	Female	165	72.0 [66.5–77.5]	
BMI (kg/m^2^)	Male	228	28.63 [25.24–32.70]	>0.05
	Female	156	28.17 [24.68–32.61]	

**Table 3:** Comparative statistical analysis of the anthropometric characteristics of the patients in each period.

Variables	Period	n	Median [IQR]	*p*
Age (years)	2019	202	68.5 [63.0–76.25]	0.605
	2021	101	67.0 [61.0–75.0]	
	2023	103	70.0 [63.0–76.0]	
BMI (kg/m^2^)	2019	192	28.21 [24.74–32.02]	0.183
	2021	90	28.1 [25.08–32.87]	
	2023	91	29.5 [25.32–35.48]	

**Table 4:** Analysis of heart failure categories regarding NYHA class and LVEF value in the entire cohort.

Variables	Categories				*p*
NYHA class	I	II	III	IV	<0.01
n	3	128	227	47	
Percentage (%)	0.74%	31.6%	56.04%	11.6%	
LVEF class	HFpEF	HFmrEF	HFrEF		<0.01
n	200	47	121		
Percentage (%)	54.35%	12.77%	32.88%		

**Table 5:** NYHA classes’ distribution and analysis in relation to gender, environment of origin, type of admission, and periods.

Year	Variables	NYHA Class						
		I	II	* *p*	III	* *p*	IV	* *p*
2019	Female	0	26	0.0833	51	0.148	8	0.685
	Male	1	51		57		8	
	Rural	0	43	0.956	57	0.601	11	0.371
	Urban	1	34		51		5	
	Appointment	1	65	0.0112	76	0.310	7	0.0108
	Emergency	0	12		32		9	
2021	Female	1	6	1.00	13	0.104	12	0.167
	Male	0	12		41		15	
	Rural	0	10	0.654	27	0.816	11	0.510
	Urban	1	8		27		16	
	Appointment	0	15	** 0.0093	35	0.0673	6	0.0001
	Emergency	1	3		19		21	
2023	Female	1	12	0.311	32	0.366	2	** 1.0
	Male	0	21		33		2	
	Rural	1	12	0.458	30	0.544	2	** 1.0
	Urban	0	21		35		2	
	Appointment	1	32	** 0.097	57	1.0	1	** 0.0048
	Emergency	0	1		8		3	

* Chi square test, ** Fisher test.

**Table 6:** LVEF analysis in relation to gender throughout the years.

Year	Variables	Median [IQR]	Gender Comparison *p* Value	Male Comparison *p* Value	Female Comparison *p* Value
	Gender				
2019	Male	50 [40.0–55.0]	0.0619	2019 vs. 2021	
	Female	50 [48.38–55.0]		* 0.057	* 0.054
2021	Male	45 [30.0–50.0]	0.766	2021 vs. 2023	
	Female	45 [25.0–52.5]		* 3.0	* 0.421
2023	Male	45 [35.5–50.0]	0.0116	2019 vs. 2023	
	Female	50 [40.0–54.0]		* 0.421	* 3.0
	**Admission Type**			**Appointment Comparison *p* Value**	**Emergency Comparison *p* Value**
2019	Appointment	50 [45.0–55.0]		2019 vs. 2021	
	Emergency	45 [35.0–50.0]		* 0.007	* 0.230
2021	Appointment	50 [31.25–50.0]		2021 vs. 2023	
	Emergency	40 [25.0–50.0]		* 0.107	* 0.310
2023	Appointment	50 [40.0–52.0]		2019 vs. 2023	
	Emergency	40 [35.0–40.0]		* 0.668	* 1.0

* Bonferroni correction applied.

**Table 7:** Comparative analysis of length of hospitalization by year.

Year	Variable	Median [IQR] (Days)
2019	Total	7.0 [5.0–9.25]
	Male	7.0 [4.0–8.0]
	Female	7.0 [6.0–10.0]
2021	Total	8.0 [6.0–12.0]
	Male	8.0 [6.0–12.0]
	Female	9.0 [6.0–13.0]
2023	Total	7.0 [5.0–10.0]
	Male	6.5 [4.0–9.25]
	Female	7.0 [5.5–9.5]

**Table 8:** Statistical analysis of NYHA classes in relation to length of stay in hospital during each period.

Year	NYHA Class	Length of Stay in Hospital		* *p*	** *r*	** *p*	*** *β*_1_	*** *p*
		Median (Days)	n					
2019	I	3.0	1	<0.001	0.170	0.014	0.5	0.289
	II	7.0	77					
	III	7.0	108					
	IV	14.0	16					
2021	I	6.0	1	0.0053	0.360	<0.001	2.0	0.01
	II	7.0	18					
	III	8.0	54					
	IV	12.0	27					
2023	I	6.0	1	<0.001	0.420	<0.001	3.0	<0.001
	II	5.0	33					
	III	7.0	65					
	IV	19.0	4					

* Kruskal–Wallis test, ** Spearman test, *** quantile regression; *r*—regression coefficient; *β***_1_**—regression coefficient that represents the estimated change in the median length of hospital stay for each one-unit increase in NYHA class.

**Table 9:** Statistical analysis of associated diseases and death rate during hospitalization.

Associated Disease	Category	Deceased During Hospitalization		*p*	Odds Ratio (OR)	Confidence Interval (CI = 95%)
Valvular heart disease (VHD)		Yes	No	<0.001	0.0855	0.02–0.366
	Yes	14	382			
	No	3	7			

Other associated diseases did not reach the statistical significance threshold (*p* > 0.05).

**Table 10:** Statistical analysis regarding the associated diseases each year.

Associated Disease	2019 (n)	2021 (n)	2023 (n)	*p*
Angina pectoris	42	14	10	0.0347
Ventricular or supraventricular arrhythmia (VA or SVA)	83	49	51	0.272
Chronic obliterating arteriopathy of the lower limbs (COALL)	17	15	11	0.229
Chronic venous disease	54	18	30	0.134
Atherosclerosis	59	20	39	0.0177
Right bundle branch block (RBBB)	24	33	19	<0.001
Left bundle branch block (LBBB)	21	39	27	<0.001
Arterial hypertension (HTN)	173	80	87	0.349
Atrial fibrillation/Atrial flutter (AF)	100	60	60	0.167
Pulmonary hypertension (PH)	120	58	44	0.0176
History of Myocardial Infarction (MI)	43	28	17	0.148
Ischaemic heart disease (IHD)	122	62	52	0.188
Atrioventricular conduction disorders	17	14	6	0.121
Valvular heart disease (VHD)	198	97	101	0.533

**Table 11:** Statistical analysis of cardiovascular associated diseases by gender.

Associated Disease	Gender	2019 (n)	2021 (n)	2023 (n)	*p*
Angina Pectoris	Male	23	10	4	0.100
	Female	19	4	6	0.001
Ventricular or supraventricular arrhythmia	Male	53	37	29	0.449
	Female	30	12	22	0.0221
Chronic obliterating arteriopathy of the lower limbs (COALL)	Male	9	15	8	0.0205
	Female	8	0	3	0.0116
Atherosclerosis	Male	34	14	22	0.073
	Female	25	6	17	<0.01
Right bundle branch block (RBBB)	Male	13	10	11	<0.001
	Female	11	4	1	0.481
Left bundle branch block (LBBB)	Male	15	24	13	<0.001
	Female	6	9	6	0.337
Chronic venous disease	Male	28	28	21	0.347
	Female	26	11	6	<0.01
Arterial hypertension (HTN)	Male	95	7	7	<0.001
	Female	78	8	12	<0.001
Atrial fibrillation/Atrial flutter (AF)	Male	54	13	4	0.066
	Female	46	10	13	<0.001
Pulmonary hypertension (PH)	Male	69	43	32	0.120
	Female	51	17	28	<0.001
History of Myocardial Infarction (MI)	Male	25	14	16	0.372
	Female	18	3	2	0.320
Ischaemic heart disease (IHD)	Male	71	39	24	0.074
	Female	51	19	20	<0.001
Atrioventricular conduction disorders	Male	11	13	7	0.109
	Female	6	15	10	0.751
Valvular heart disease (VHD)	Male	116	40	24	0.421
	Female	82	22	28	<0.001

**Table 12:** Statistical analysis of associated diseases or behaviours (other than cardiovascular).

Disease/Behaviour	Variable	2019 (n)	2021 (n)	2023 (n)	*p*
Anemia	Total	36	25	24	0.298
	Male	15	11	14	0.130
	Female	21	14	10	0.126
Chronic kidney disease (CKD)	Total	70	40	37	0.697
	Male	35	24	19	0.722
	Female	35	16	18	0.0087
Endocrine diseases	Total	26	15	19	0.430
	Male	12	24	19	0.894
	Female	14	16	18	0.438
Chronic obstructive pulmonary disease (COPD)	Total	33	14	12	0.534
	Male	26	10	11	0.461
	Female	7	4	1	0.105
Smoker	Total	34	17	18	0.988
	Male	24	29	45	0.450
	Female	10	31	42	0.0224
Diabetes mellitus (type 2)	Total	57	42	38	0.05
	Male	34	24	22	0.959
	Female	23	18	16	0.884

**Table 13:** Statistical comparative analysis of NT-proBNP values throughout the periods and in relation to other parameters.

NT-proBNP Median Values (pg/mL)						
Variables	2019	*p*	2021	*p*	2023	*p*
Female	3983	0.033	2747.3	0.304	1686	0.177
Male	2132		2962		665.05	
Rural	3983	0.109	2480.65	0.373	1846.13	0.031
Urban	2413		3748		533.00	
Appointment	407.3	<0.001	1126.57	<0.001	899.4	0.0307
Emergency	5464		5572		5345.55	
* Appointment 2019 vs. 2021	** *p* = 0.0162					
Under 75 years old	1214	<0.001	2212.75	0.054	522.13	0.015
Over 75 years old	6653.5		3748		1954.81	

Data are presented as median values; * Other comparison did not show a significant difference; ** Bonferroni correction applied.

**Table 14:** Statistical analysis between NT-proBNP values and LVEF through linear regression.

Year	log (NT-proBNP) Coefficient	*p*
2019	−2.145	0.0296
2021	−3.389	<0.001
2023	−1.688	0.0119

**Table 15:** The analysis of mortality rates.

	In-Hospital Mortality			*p*
	Male	Female	Total	
2019	6.84%	9.41%	3.96%	0.201
2021	10.29%	21.21%	13.86%	
2023	3.57%	4.26%	3.88%	

## Data Availability

The data presented in this study are available on request from the first author and corresponding author due to ethical reasons.

## Abbreviations

The following abbreviations are used in this manuscript:

ACE-2	Angiotensin converting enzyme 2
AF	Atrial fibrillation
APN	N-aminopeptidase
ARDS	Acute Respiratory Distress Syndrome
BMI	Body mass index
CK	Creatine kinase
CKD	Chronic kidney disease
COALL	Chronic obliterans arteriopathy of the lower limb
COPD	Chronic obstructive pulmonary disease
COVID-19	Coronavirus disease 2019
CRHF	Chronic right heart failure
CVD	Cardiovascular diseases
DDP4	4-dieptidyl peptidase
HF	Heart failure
HFmrEF	Heart Failure with mildly reduced ejection fraction
HFpEF	Heart Failure with preserved ejection fraction
HFrEF	Heart Failure with reduced ejection fraction
HTN	Hypertension
IHD	Ischemic heart disease
LBBB	Left bundle branch block
LVEF	Left ventricular ejection fraction
MERS	Middle East Respiratory Syndrome
MI	Myocardial infarction
NT-proBNP	N-terminal pro-B-type natriuretic peptide
NYHA	New York Heart Association
PH	Pulmonary hypertension
RBBB	Right bundle branch block
RHF	Right heart failure
SARS	Severe Acute Respiratory Syndrome
SARS-CoV-2	Severe acute respiratory syndrome coronavirus 2
WHO	World Health Organization
